# Antibiotic sensitivity pattern of gram negative bacilli isolated from the lower respiratory tract of ventilated patients in the intensive care unit

**DOI:** 10.4103/0972-5229.58540

**Published:** 2009

**Authors:** Nidhi Goel, Uma Chaudhary, Ritu Aggarwal, Kiran Bala

**Affiliations:** **From:** Department of Microbiology, Pt. B. D. Sharma University of Health Sciences, Rohtak, India

**Keywords:** Antibiotic susceptibility, gram negative bacilli, intensive care unit, ventilated patients

## Abstract

**Background:**

Lower respiratory tract infections (LRTIs) are the most frequent infections among patients in Intensive care units (ICUs).

**Aims:**

To know the bacterial profile and determine the antibiotic susceptibility pattern of the lower respiratory tract isolates from patients admitted to the ICU.

**Settings and Design:**

Tertiary care hospital, retrospective study.

**Materials and Methods:**

Transtracheal or bronchial aspirates from 207 patients admitted to the ICU were cultured, identified, and antibiotic sensitivity was performed by standard methods.

**Statistical Analysis Used:**

SPSS software was used for calculation of % R of 95% confidence interval (CI).

**Results:**

Of 207 specimens, 144 (69.5%) were culture positive and 63 (30.4%) showed no growth. From 144 culture positives, 161 isolates were recovered, of which 154 (95.6%) were Gram negative bacilli (GNB). In 17 (11.0%) patients, two isolates per specimen were recovered. The most common GNB in order of frequency were *Pseudomonas aeruginosa* (35%), *Acinetobacter baumannii* (23.6%), and *Klebsiella pneumoniae* (13.6%). A very high rate of resistance (80-100%) was observed among predominant GNB to ciprofloxacin, ceftazidime, co-trimoxazole, and amoxycillin/clavulanic acid combination. Least resistance was noted to meropenem and doxycycline.

**Conclusion:**

Nonfermenters are the most common etiological agents of LRTIs in ICU. There is an alarmingly high rate of resistance to cephalosporin and β-lactam-β-lactamase inhibitor group of drugs. Meropenem was found to be the most sensitive drug against all GNB. *Acinetobacter* and *Klebsiella* spp. showed good sensitivity to doxycycline.

## Introduction

Lower respiratory tract infections (LRTI) are the most common bacterial infections among patients in intensive care units (ICUs) occurring in 10-25% of all ICU patients and resulting in high overall mortality, which may range from 22-71%.[1,2] Most common bacterial agents of LRTI in the ICU are *Pseudomonas*, *Acinetobacter*, *Klebsiella*, *Citrobacter*, *Escherichia coli*.[3–5] In almost all cases, there is a need to initiate empirical antimicrobial treatment before obtaining the microbial results, but the situation is further complicated by the emergence of multiple beta lactamase producers and multidrug resistant pathogens. In a recent report, Infectious Disease Society of America, specifically addressed three categories of gram negative bacilli (GNB), namely extended spectrum beta lactamase (ESBL) producing *Escherichia coli*, and *Klebsiella* spp., Multidrug resistant (MDR) *Pseudomonas*, and carbapenem resistant *Acinetobacter* spp., as high priority bacterial pathogens.[6] All these major reports indicate the need for obtaining data on prevalent strains in the ICU along with the susceptibility pattern, to help in revising antibiotic policy and guiding clinicians for the better management of patients. Prevalent flora and antimicrobial resistance pattern may vary from region to region depending upon the antibiotic pressure in that locality. Therefore, the present study was designed to know the bacterial profile and determine the antimicrobial resistance pattern among the aerobic GNB isolated from LRT of patients admitted to the ICU of our institute.

## Materials and Methods

The present retrospective study was conducted in the Microbiology department of a teaching tertiary care hospital during Jan-Dec 2007. Transtracheal or bronchoscopic aspirates collected aseptically from 207 patients of all age and sex groups requiring mechanical ventilation for at least three days were included in study. All samples were plated right after the collection and were further processed as per standard protocol.[7] Single or mixed growth (two or more than two isolates per specimen) isolated from all the eligible consecutive samples were identified by observing the colony characteristic on the blood, Mac-Conkey agar plate and biochemical reactions using standard microbiological methods.[7] Isolates from repeat culture of previously recruited patients and isolates identified as commensals or contaminants were excluded. Susceptibility testing was done by Disc diffusion method.[8] The following antibiotics (Hi-Media Disc in mcg) were tested: Amikacin(Ak) (30), ciprofloxacin(Cf) (5), ofloxacin (Of) (5), aztreonam (Ao) (30), netilmicin (Nt) (30), doxycycline (Do) (30), cotrimoxazole (co) (25), ceftazidime (Cz) (30), ceftizoxime (Ck) (30), meropenem (Mr) (10), amoxycillin/clavulanic acid (Ac) (20/10), piperacillin/tazobactum (TZP) (100/10). Zone diameter was measured and interpreted as per the Clinical and Laboratory Standards Institute (CLSI) guidelines. For quality control of disc diffusion tests ATCC control strains of *E. coli* ATCC 25922, *S*. *aureus* ATCC 25923 and *P*. *aeruginosa* ATCC 27853 strains were used.

### Statistical analysis

For retrospective analysis, SPSS software was used for calculation of percentage resistance of 95% confidence interval (CI).

## Results

During the study period, laboratory data of 207 patients whose LRT specimens were received in our laboratory was evaluated. Male to female ratio was 1.8:1. Out of the 207 patients, 70 were from the surgical ward, 62 from urology, 35 from medicine, 28 from nephrology, 12 from neuromedicine. Out of 207 specimens, 144 (69.5%) were culture positive whereas, 63 (30.43%) specimens showed no growth. From the 144 culture positive specimens, 161 isolates were recovered. Out of 161 isolates, 154 (95.6%) were GNB, three (1.86%) were *Candida* spp., and four (2.4%) were Gram positive cocci. In 17 (11.0%) specimens, there were two isolates per specimen and 127 (82.4%) specimens showed growth of a single organism. Table 1 represents the distribution of micro organisms recovered from the LRT specimens of ICU patients. The most common GNB in order of frequency were *P*. *aeruginosa* (35%), *Acinetobacter baumannii* (23.6%) and *Klebsiella pneumoniae* (13.6%). Very high rate of resistance (60-100%) was observed among *A*. *baumannii* and *K*. *pneumoniae* isolates to ceftazidime, amoxyclav, ciprofloxacin, amikacin, and cotrimoxazole. Meropenem and doxycycline were the most effective *in vitro* drugs against *A*. *baumannii*, *K*. *pneumoniae*, and *Enterobacter* [Table 2]. *P*. *aeruginosa* isolates showed high rate of resistance to aztreonam (94.7%), netilmicin (70.2%), ceftazidime (68.4%), ceftizoxime (68.4%), and ofloxacin (68.4%) [Table 2]. Out of 57 isolates of *P*. *aeruginosa*, 23 (40%) were resistant to all the antibiotics used against *P*. *aeruginosa* in the panel. Meropenem was the most effective (77.2%) drug *in vitro* followed by piperacillin/ tazobactum combination (50.5%) [Table 2]. Bacterial resistance rates (%R 95% CI) to doxycycline for various isolates except *P*. *aeruginosa* are given in Table 3.

## Discussion

Pneumonia is a frequent complication in patients admitted to the ICU. It is frequently polymicrobial with predominently multi drug resistant GNB, such as *A*. *baumannii*, *P*. *aeruginosa*, *K*. *pneumoniae*, *E*. *coli*.[7,9,10] In our study, 97.4% isolates were GNB. *P*. *aeruginosa* (35%) being the most common isolate followed by *A*. *baumannii* (23.6%) and *K. pneumoniae*. In 10.75% cases, two isolates were recovered from a single specimen, in contrast to the other study that reported two to three isolates per specimen in 16.3% cases.[5] Antibiotic resistance is a major problem in ICU admitted patients. We noticed 100%, 96.9%, and 68.4% resistance to ceftazidime against *A*. *baumannii*, *Klebsiella* spp. and *P*. *aeruginosa*, respectively. Similar observations were made by other investigators that reported 96-100% resistance;[11,12] whereas, other workers have reported lower rate of resistance (37-67.5%) to ceftazidime.[11,13] High rate of resistance at our center might be due to the selective influence of extensive usage of third generation cephalosporins. Carbapenems are frequently used as a last choice in treating serious infections caused by GNB. In our study, 25.6% isolates of *Acinetobacter* spp., 22.8% isolates of *P*. *aeruginosa*, and 9% isolates of *Klebsiella* spp., were resistant to meropenem in contrast to another study, where meropenem resistance was found in 14.2% isolates of *A*. *baumannii* and 12-42.5% isolates of *P*. *aeruginosa*, respectively.[4,14] Another study reported 100% sensitivity to meropenem against *Klebsiella* spp.[15] This finding suggests that meropenem should be used judiciously in ventilated patients to prevent any further increase in resistance to meropenem. Another important observation of our study was that doxycycline, an old drug not included in empirical treatment in ICUs, nowadays had shown good sensitivity against *A*. *baumannii*, *Klebsiella* spp., *Enterobacter* spp., *E. coil,* and *Citrobacter* spp. [Table 3]. Vila *et al*., reported 98% sensitivity to doxycycline among *A*. *baumannii*.[16] We observed that 33% isolates of *A*. *baumannii* were sensitive and 64.4% strains were intermediate sensitive (IS) to doxycycline. Intermediate sensitive strains are amenable to treatment when large doses of the drug are used.[17] We did not perform MIC of IS strains, since this is a retrospective analysis of laboratory data and strains were not preserved for further study. Further studies are required to evaluate the usefulness of doxycycline for ICU admitted patients.

**Table 1:** Different microorganisms isolated from the lower respiratory tract specimen from intensive care unit patients

Organism	Bronchioalveolr lavage (%)	Tracheal aspirate (%)	Total (%)
*P. aeruginosa*	13	44	57 (35)
*A. baumannii*	1	37	38 (23.6)
*K. pneumoniae*	1	21	22 (13.6)
*Enterobacter spp.*	0	17	17 (10.5)
*E. coli*	0	12	12 (7.4)
*C. freundii*	0	8	8 (4.9)
*S. aureus*	0	3	3 (1.8)
*Coagulase negative*			
*S. aureus*	0	1	1 (0.6)
*Candida spp.*	0	3	3 (1.8)
*Total*	15 (9.3)	146 (90)	161

**Table 2:** Antimicrobial resistance rates (%) for the gram negative bacilli recovered from lower respiratory tract secretions of intensive care unit patients

*Antimicrobials*	*A. baumannii* (38)	*K. pneumoniae* (22)	*C. freundii* (8)	*E. coli* (12)	*Enterobacter* (17)	*P. aeruginosa* (57)
Cotrimoxazole	79.5	90.9	100	100	100	ND*
Doxycycline	2.6	9.1	0 0	0	ND
Amikacin	87.2	63.6	75	41.7	72.2	61.4
Netilmicin	ND	ND	ND	ND	ND	70.2
Ciprofloxacin	89.7	95.5	100	100	100	ND
Ofloxacin	ND	ND	ND	ND	ND	68.4
Ceftizoxime	ND	ND	ND	ND	ND	68.4
Ceftazidime	100	96.9	87.5	91.7	100	68.4
Meropenam	25.6	9.1	12.5	0	11.1	22.8
Amoxyclav	97.4	100	100	91.7	100	ND
Ticarcillin/Tazobactam	ND	ND	ND	ND	ND	49.1
Aztreonam	ND	ND	ND	ND	ND	94.7

* ND - Not Done

**Table 3:** Bacterial resistance rates (%R 95%CI) to doxycycline for various isolates recovered from lower respiratory tract specimens

Organism(n)	%R*	%IS**	%S***	%R 95%CI
*A. baumannii* (38)	2.6	64.1	33.3	0.1-15.1
*C. freundii* (8)	0	75	25	0.0-40.2
*Enterobacter *spp. (17)	0	94.4	5.6	0.0-21.9
*E. coli* (12)	0	83.3	16.7	0.0-30.1
*K. pneumoniae* (22)	9.1	77.7	13.6	1.6-30.6

* % R - Percentage resistance

** % IS - Percentage intermediate sensitive

***% S - Percentage sensitive

## Limits and outcome

One of the potential limitations of this study is that epidemiologic analysis, ESBL phenotypic detection and MIC of doxycycline was not carried out. Despite this limitation our data can be used for local therapeutic choices.

We conclude that nonfermenters are the most common etiological agents of LRTIs in ICU. There is an alarmingly high rate of resistance to cephalosporins, β lactam-β-lactamase inhibitors, and carbapenem against predominant organisms. We suggest that further studies should be carried out to evaluate the usefulness of doxycycline against the ICU pathogens. Judicious use of older and newer antimicrobial agents is essential to prevent the emergence of multi drug resistant bacteria in the ICU.
